# Prognostic impact of body temperature trajectories from emergency department arrival to intensive care unit admission – insights from the TraumaRegister DGU^®^

**DOI:** 10.1186/s13049-026-01642-0

**Published:** 2026-06-10

**Authors:** Jan Stein, Friederike Weidemann, Rolf Lefering, Thomas A. Schildhauer, Oliver Cruciger, Uwe Hamsen, the TraumaRegister DGU

**Affiliations:** 1 Department of General and Trauma Surgery, BG University Hospital Bergmannsheil, Ruhr University Bochum, Bürkle De La Camp Platz 1, 44789 Bochum, Germany; 2 Department of Trauma Surgery, Hannover Medical School, Hannover, Germany; 3 Department of Intensive Care Medicine, HELIOS Medical Center Schwerin, University Campus of MSH Medical School Hamburg, Schwerin, Germany; 4 Institute for Research in Operative Medicine (IFOM), University Witten/Herdecke, Witten/Herdecke, Germany; 5 Committee on Emergency Medicine, Intensive Care and Trauma Management (Sektion NIS) of the German Trauma Society (DGU), Munich, Germany

## Abstract

**Background:**

Hypothermia is a significant and independent predictor of mortality in patients with severe trauma and constitutes a key component of the trauma-related lethal triad, along with acidosis and coagulopathy. While the prognostic impact of hypothermia at isolated time points is well established, the clinical relevance of dynamic body temperature changes during the early in-hospital phase remains poorly understood. This study aims to evaluate the association between body temperature trajectories from emergency department (ED) admission to intensive care unit (ICU) transfer and clinical outcomes in major trauma patients.

**Methods:**

Retrospective data from the TraumaRegister DGU^®^ (TR-DGU) for the period 2015–2023 were analysed. Inclusion criteria comprised an Injury Severity Score (ISS) ≥ 9, ICU admission following ED care, and documented core body temperatures at both ED and ICU. Hypothermia was defined as a body temperature ≤ 35 °C. Patients were stratified into four groups based on the presence or absence of hypothermia at each time point. Further subgroup analysis assessed temperature trends (decreasing vs. stable/increasing) and their associations with mortality, coagulopathy, transfusion requirements, and sex distribution.

**Results:**

A total of 34,877 patients met the inclusion criteria (mean age 54.2 ± 20.9 years; 71.6% male; mean ISS 22.8 ± 11.5). Hypothermia was present in 10.7% upon ED admission and 9.7% of patients at ICU transfer. Persistent hypothermia was associated with the highest mortality (43.5%), coagulopathy (28.2%), and transfusion rates (31.8%). New onset of hypothermia in the ICU was also linked to poor prognosis (mortality 31.9%). Patients with a temperature decrease (11.2%) had significantly higher injury severity and mortality compared to those with stable or rising temperatures. Female patients were disproportionately represented in hypothermic ICU groups (38%).

**Conclusion:**

Hypothermia—particularly when persistent or newly acquired—emerges as a strong predictor of adverse outcomes in trauma patients. Dynamic temperature monitoring between ED and ICU provides critical prognostic insights that extend beyond static measurements. Early identification and management of thermal dysregulation, including sex-specific considerations, should be integral components of contemporary trauma care protocols.

## Background

Hypothermia is a well-recognized and independent determinant of mortality in patients suffering from severe traumatic injuries, with reported fatality rates ranging from 25% to 40% [1–3]. Within the context of trauma care, it forms—alongside acidosis and coagulopathy—the pathophysiological triad commonly referred to as the “lethal triad of trauma”. These three factors interact synergistically, amplifying one another’s effects in a self-perpetuating cycle of physiological decline that can rapidly progress to multi-organ failure and death [4, 5].

Despite its clinical importance, the definition of hypothermia varies across studies. Nonetheless, most investigations adopt a body temperature of ≤ 35 °C as the diagnostic threshold [3, 6, 7]. Hypothermia is typically further stratified by severity into mild (35–32 °C), moderate (32–28 °C), and severe (< 28 °C) categories [3, 8, 9], reflecting its graded impact on hemostasis, metabolism, and cardiovascular function.

In trauma patients, hypothermia is not confined to the prehospital phase or initial ED presentation. Rather, it constitutes a persistent challenge that extends throughout the continuum of early care—from prehospital management and ED resuscitation to intraoperative intervention and subsequent ICU admission [6, 9]. While prior research has elucidated the prognostic significance of hypothermia at specific time points, the clinical implications of dynamic temperature trajectories during the early in-hospital phase remain inadequately understood.

Against this background, the present study aims to investigate the prognostic relevance of body temperature trends between ED admission and ICU transfer in patients with major trauma, with particular focus on their association with adverse clinical outcomes.

## Materials and methods

### Study design

A registry evaluation of the TR-DGU of the German Trauma Society (Deutsche Gesellschaft für Unfallchirurgie) was conducted for the period from January 2015 to December 2023. The study is registered under TR-DGU ID 2024-042.

The TR-DGU was founded in 1993. The aim of this multicentre database is a pseudonymised and standardised documentation of severely injured patients. Data are collected prospectively in four consecutive time phases from the site of the accident until discharge from hospital: (A) Pre-hospital phase, (B) ED and initial surgery, (C) ICU and (D) Discharge. The documentation includes detailed information on demographics, injury pattern, comorbidities, pre- and in-hospital management, course in the ICU, relevant laboratory findings including data on transfusion and outcome of each individual. The inclusion criterion is admission to hospital via emergency room with subsequent ICU/ICM care or reach the hospital with vital signs and die before admission to ICU. The infrastructure for documentation, data management, and data analysis is provided by AUC – Academy for Trauma Surgery (AUC - Akademie der Unfallchirurgie GmbH), a company affiliated with the German Trauma Society. The scientific leadership is provided by the Committee on Emergency Medicine, Intensive Care and Trauma Management (Sektion NIS) of the German Trauma Society. The participating hospitals submit their data pseudonymised into a central database via a web-based application. Scientific data analysis is approved according to a peer review procedure laid down in the publication guideline of TR-DGU.

The participating hospitals are primarily located in Germany (90%), but a rising number of hospitals of other countries contribute data as well (at the moment from Austria, Belgium, China, Finland, Luxembourg, Slovenia, Switzerland, the Netherlands, and the United Arab Emirates). Currently, more than 31,000 cases from around 700 hospitals are entered into the database per year. Participation in TR-DGU is voluntary. For hospitals associated with TraumaNetzwerk DGU^®^, however, the entry of at least a basic data set is obligatory for reasons of quality assurance.

### Study group

During the study period, a total of 358,681 trauma cases were documented in the TR-DGU. To ensure structural comparability of patient management, the analysis was restricted to data from Germany, Austria, and Switzerland (GER/A/CH). Cases documented with the short form of the TR-DGU questionnaire were excluded due to missing temperature data.

Major trauma was defined as the presence of at least one injury with an Abbreviated Injury Scale (AIS) score ≥ 3, resulting in an ISS of ≥ 9 [10]. Only patients who were primarily admitted directly from the scene of injury to a participating hospital were considered eligible.

Exclusion criteria comprised incomplete documentation of body temperature at either ED or ICU admission, early transfer to another hospital (final outcome unknown), as well as patient age < 16 years.

**Fig. 1:**
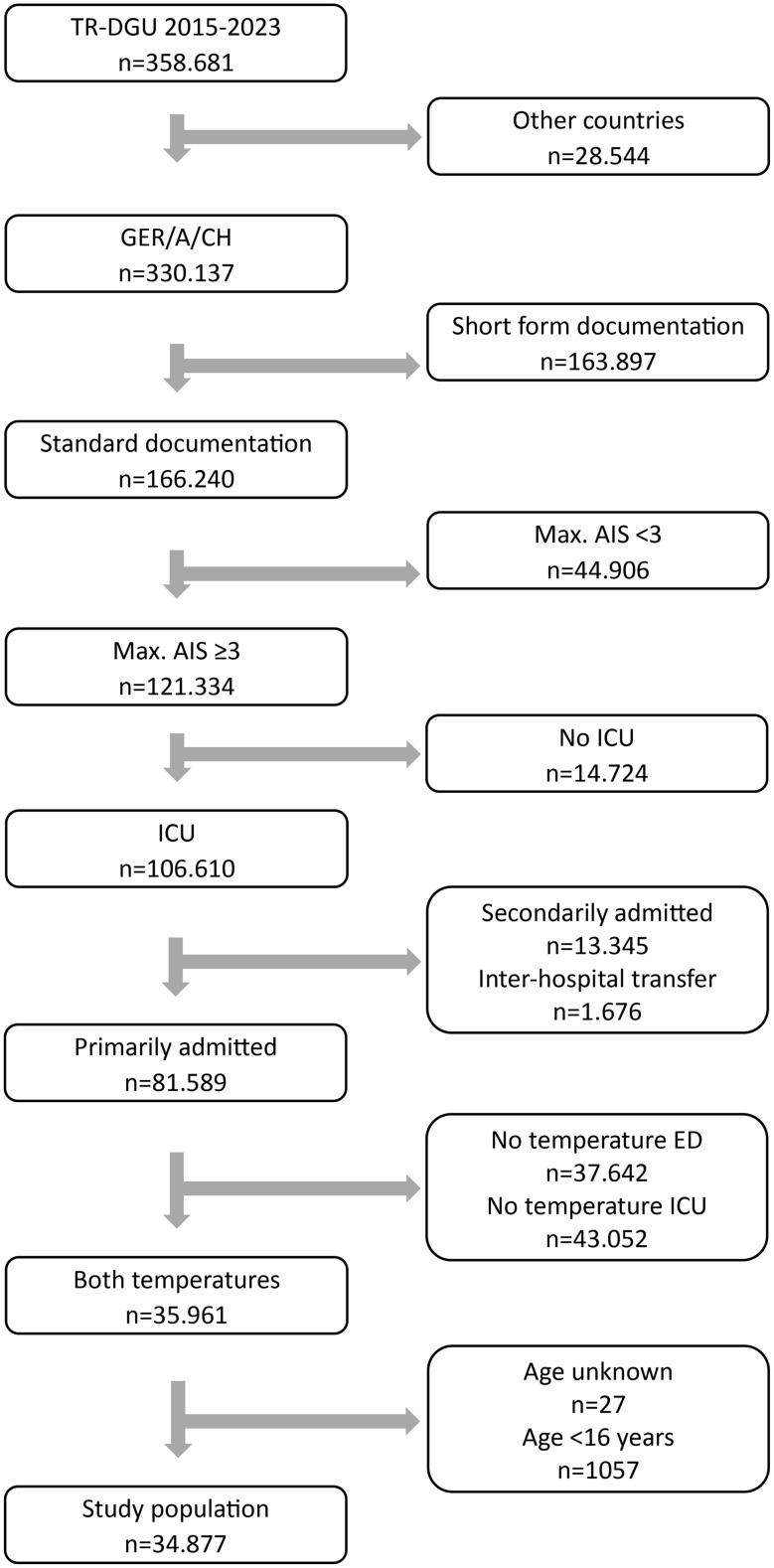
Flow chart, illustrating the patient selection process; *TR-DGU* TraumaRegister DGU^®^, *GER/A/CH* Germany, Austria, and Switzerland, *AIS* Abbreviated Injury Scale, *ICU* intensive care unit

A detailed overview of the case selection and stratification process is shown in Fig. 1.

Hypothermia was defined as a body temperature of ≤ 35 °C. Within the TR-DGU, only body temperature at ED and ICU admission is recorded, without specification of the measurement method. Coagulopathy was identified based on at least one of the following criteria: partial thromboplastin time (PTT) ≥ 40 s, or Quick’s value ≤ 60%, or international normalized ratio (INR) ≥ 1.4.

To maintain consistency in timing-related variables, ICU admission was categorized by pathway, distinguishing between direct admission and admission after surgical intervention in the operating room (OR).

In addition, data were analysed in relation to demographic and clinical variables, including age, sex, the presence of severe traumatic brain injury (defined as AIS_head_ ≥ 3), and the administration of blood transfusions.

### Statistics

Statistical analysis was performed using SPSS (version 29, IBM Inc., Armonk, NY, USA). Continuous variables were presented as mean with standard deviation (SD); time intervals were presented as median with quartiles. Categorical variables (e.g., presence of hypothermia, sex distribution, injury severity, transfusion rates, and mortality) were presented as absolute numbers and percentages. The presence of hypothermia was analysed at two distinct time points: arrival in the ED and admission to the ICU using a cross-tabulation.

All statistical analyses were exploratory and hypothesis-generating. No adjustment for multiple testing was performed; therefore, p-values should be interpreted descriptively. Only selected comparisons were formally evaluated with a statistical test (chi-squared test for frequencies; Mann-Whitney U-test for metric data).

## Results

A total of 34,877 trauma patients from 238 hospitals met the inclusion criteria and were included in the final analysis. The mean age of the study population was 54.2 ± 20.9 years, with a predominance of male patients (71.6%). The mean ISS was 22.8 ± 11.5, with a median of 21. Overall in-hospital mortality was 13.5% (*n* = 4,723).

The median time between ED admission and ICU transfer was 141 (70–256) minutes. In patients directly transferred from the ED to the ICU, the median interval was 83 (55–138) minutes. In contrast, patients admitted via the OR experienced substantially longer transfer times, with a median of 266 (200–360) minutes.

Temperature data revealed that 10.7% of patients were hypothermic (≤ 35.0 °C) upon ED admission, while 9.7% were hypothermic at ICU admission. Hypothermia at either time point was associated with elevated mortality rates, rising from 28.1% at ED presentation to 36.6% at ICU admission. The mortality rate among patients hypothermic at both time points was 43.5%. Coagulopathy was present in 25.5% of patients presenting with hypothermia upon ED admission, and in 27.6% with hypothermia on ICU admission.

In contrast, normothermic patients demonstrated the most favorable outcomes, with the lowest observed mortality (10.4% at ED admission; 9.3% at ICU admission) and coagulopathy rates. Hyperthermia (≥ 38.0 °C) was rare, occurring in 1.2% of patients at ED admission and 3.0% at ICU admission. Although hyperthermic patients also exhibited increased mortality (17.0% at ED; 14.1% at ICU), their coagulopathy rate (15.7% at ED; 12.2% at ICU) remained lower than that of hypothermic individuals.

A detailed breakdown of body temperature categories, along with corresponding rates of coagulopathy and in-hospital mortality, is presented in Table 1.

**Table 1:** Association of body temperature on ED and ICU admission with coagulopathy and mortality

Temperature Range (°C)^a^	*n* (%)	Coagulopathy (%)^b^	Mortality (%)
**ED admission**			
≤ 35.0	3739 (10.7%)	25.5	28.1
35.1–35.9	5808 (16.7%)	16.0	16.0
36.0–36.9	16,750 (48.0%)	11.3	10.9
37.0–37.9	8169 (23.4%)	11.2	10.4
≥ 38.0	411 (1.2%)	15.7	17.0
**ICU admission**			
≤ 35.0	3397 (9.7%)	27.6	36.6
35.1–35.9	4078 (11.7%)	17.2	19.6
36.0–36.9	13,348 (38.2%)	11.8	10.0
37.0–37.9	13,002 (37.3%)	11.0	9.3
≥ 38.0	1052 (3.0%)	12.2	14.1

*ED* emergency department, *ICU* intensive care unit

^a^ hypothermia definition: body temperature ≤ 35.0 °C

^b^ coagulopathy definition: partial thromboplastin time ≥ 40 s, or Quick’s value ≤ 60%, or international normalized ratio ≥ 1.4

To elucidate the prognostic implications of temporal temperature dynamics, patients were stratified into four groups based on the presence or absence of hypothermia at ED and ICU admission. The majority of patients (83.2%, *n* = 29,033) remained normothermic throughout the early treatment course (Group 1). New-onset hypothermia upon ICU admission was observed in 6.0% (*n* = 2,105; Group 2). In 7.0% of cases (*n* = 2,447), hypothermia was present upon ED arrival but had resolved prior to ICU transfer (Group 3), while 3.7% (*n* = 1,292) remained hypothermic at both time points (Group 4).

Patients in Group 4—those with persistent hypothermia—exhibited the most severe clinical profiles, including the highest rate of severe traumatic brain injury (47.0%), the highest in-hospital mortality (43.5%), and the greatest need for blood transfusion in the ED (31.8%). This group also had the highest median age (60 years) and the lowest proportion of male patients (62%).

In contrast, Group 1 patients (normothermic throughout) demonstrated the most favorable outcomes, with an in-hospital mortality rate of 10.3%, a severe head injury rate of 25.5%, and transfusion requirements in 10.4% of cases.

Intermediate outcomes were observed in Group 3 (hypothermia resolved before ICU admission), which was associated with a mortality rate of 20.0% and a severe head injury rate of 37.6%. Notably, Group 2 patients—who developed hypothermia during early in-hospital care—experienced markedly worse outcomes, including a mortality rate of 31.9%, a high incidence of severe traumatic brain injury (41.7%), and substantial transfusion needs (30.8%).

A sex-specific disparity was also identified: female patients were over-represented in the hypothermic ICU groups (Groups 2 and 4, each 38%) compared to Group 1 (27%) and Group 3 (33%).

Comprehensive patient characteristics stratified by hypothermia status at ED and ICU admission are provided in Table 2.

**Table 2:** Patient characteristics stratified by the presence or absence of hypothermia at ED admission and ICU admission

Hypothermia^a^ on ED admission	No		Yes		
Hypothermia^a^ on ICU admission	No	Yes		No	Yes
	Group 1 (*n* = 29033)	Group 2 (*n* = 2105)		Group 3 (*n* = 2447)	Group 4 (*n* = 1292)
Age (mean, SD), years	53.7 (20.8)	57.1 (22.0)		54.8 (21.0)	57.8 (21.6)
Male / Female (%)	73 / 27	62 / 38		67 / 33	62 / 38
ISS (mean, SD)	21.8 (10.6)	28.2 (13.7)		27.0 (13.0)	30.2 (14.6)
Severe head injury (%)	25.5	41.7		37.6	47.0
Mortality in % (n)	10.3 (2999)	31.9 (672)		20.0 (490)	43.5 (562)
Blood transfusion in ED (%)	10.4	30.8		24.7	31.8
Time ED to ICU, (median, Q), min	135 (68–255)	173 (87–256)		173 (83–285)	142 (73–237)
Direct ICU admission (%)	33.8	47.5		46.1	39.2
ICU admission via OR (%)	61.9	48.7		49.9	57.9

*Q* quartiles, *SD* standard deviation, *ISS* Injury Severity Score, *ED* emergency department, *ICU* intensive care unit

^a^ hypothermia definition: body temperature ≤ 35.0 °C

To assess the clinical significance of early in-hospital temperature trajectories, patients were stratified based on whether core body temperature decreased or remained stable/increased between ED admission and subsequent ICU transfer. A decline in temperature was observed in 11.2% of cases (*n* = 3,901).

This subgroup exhibited markedly more severe clinical characteristics, including a higher mean ISS (26.2 ± 13.0 vs. 22.4 ± 11.2), an increased incidence of severe traumatic brain injury (37.9% vs. 26.9%, *p* < 0.001), and a substantially elevated in-hospital mortality rate (25.0% vs. 12.1%, *p* < 0.001), compared to patients with stable or rising temperatures. Moreover, this group demonstrated higher transfusion requirements in the ED (23.5% vs. 12.2%, *p* < 0.001) and was more frequently transferred to the ICU via the OR (45.2% vs. 34.9%, *p* < 0.001).

In contrast, the majority of patients (*n* = 30,976; 88.8%) maintained stable or increasing body temperatures and experienced more favorable clinical outcomes, including lower ISS, reduced transfusion needs, and significantly lower mortality rates.

A comprehensive overview of patient characteristics stratified by temperature trajectory is provided in Table 3.

**Table 3:** Patient characteristics by temperature change between ED and ICU admission

Characteristic	Stable or Increasing Temperature (*n* = 30976)	Decreasing Temperature (*n* = 3901)	Total (*n* = 34877)
Age, mean ± SD, years	54.0 ± 20.8	55.5 ± 21.7	54.2 ± 20.9
Male, n (%)	22,398 (72.3%)	2567 (65.8%)	24,965 (71.6%)
Age ≥ 65 years, n (%)	10,397 (33.6%)	1488 (38.1%)	11,885 (34.1%)
ISS, mean ± SD	22.4 ± 11.2	26.2 ± 13.0	22.8 ± 11.5
ISS ≥ 16, n (%)	22,431 (72.4%)	3138 (81.6%)	25,614 (73.4%)
Severe head injury, n (%)	8334 (26.9%)	1478 (37.9%)	9812 (28.1%)
Mortality, n (%)	3746 (12.1%)	977 (25.0%)	4723 (13.5%)
Blood transfusion in ED or OR, n (%)	3764 (12.2%)	917 (23.5%)	4681 (13.4%)
Transferred from ED to OR, n (%)	10,791 (34.9%)	1764 (45.2%)	12,555 (36.0%)
Time from ED to ICU, (median, Q), min	137 (70–256)	167 (80–260)	141 (70–256)

*SD* standard deviation, *ISS* Injury Severity Score, *Q* quartiles, *ED* emergency department, *OR* operating room, *ICU* intensive care unit

## Discussion

This study investigated the clinical relevance of dynamic body temperature changes in severely injured trauma patients between ED admission and subsequent ICU transfer. Our findings demonstrate that hypothermia—whether present upon initial presentation or acquired during early in-hospital care—is strongly associated with adverse clinical outcomes, including increased mortality, elevated rates of coagulopathy, and higher transfusion requirements. Notably, patients with persistent hypothermia throughout the ED-to-ICU interval exhibited the highest mortality (43.5%) and injury severity (ISS_mean_ 30.2), supporting previous evidence identifying hypothermia as an independent and critical prognostic factor in trauma care [1–3, 11].

Beyond isolated temperature values, the analysis of temperature trajectories revealed additional prognostic significance. Patients who developed hypothermia during early in-hospital treatment had similarly poor outcomes compared to those with persistent hypothermia, underscoring that deteriorating thermal status itself reflects clinically meaningful pathophysiology. These findings align with those of Balmer et al., who emphasised the sustained prognostic value of early temperature monitoring throughout trauma care [12]. Moreover, Erkens et al. demonstrated that admission body temperature independently predicts outcomes in critically ill patients, supporting the interpretation that hypothermia is not merely a secondary phenomenon of injury severity, but a modifiable and clinically actionable parameter [11].

Environmental and systemic conditions during the prehospital and early in-hospital phases appear to play a decisive role in the development of hypothermia. The HypoTraum study by Lapostolle et al. identified impaired consciousness, advanced age, and low ambient temperatures as key contributors to early hypothermia in trauma patients [13]. Similarly, Forristal et al. associated prolonged prehospital transport times and reduced Glasgow Coma Scale scores with an increased risk of hypothermia upon admission [14]. These observations are particularly relevant to our cohort, where patients with severe traumatic brain injury and those undergoing surgical intervention prior to ICU admission were disproportionately affected by declining temperatures.

Particular attention should be paid to patients presenting with hypothermia or developing hypothermia during early in-hospital care, as these patients more frequently sustain severe traumatic brain injury. Compared with other patient groups, these patients demonstrated a notably higher mortality rate (Table 2, Groups 2 and 4). Given the broad physiological consequences of hypothermia, it may be assumed that its adverse effects—particularly the potential aggravation of intracranial hemorrhage—could outweigh any potential neuroprotective benefits, thereby contributing to increased mortality [15]. However, based on the available registry data, the individual contribution of these factors cannot be determined.

When interpreting body temperature data, the method of temperature measurement is a critical factor [16]. Although invasive measurement via an intravascular catheter is considered the gold standard for assessing body temperature, it is not routinely applied in standard trauma patients due to its invasive nature and associated risks. Within the registry-based data collection of the TR-DGU, the specific measurement method is not documented, which precludes further differentiation. In clinical practice, tympanic infrared thermometry is commonly used due to its rapid availability in the acute setting and acceptable measuring accuracy, although some variability in measurement accuracy must be considered [17].

Our data also revealed sex-specific differences, with female patients more frequently presenting with hypothermia at ICU admission. This finding is consistent with previous research by Coleman et al., who highlighted sex-related differences in trauma physiology and resuscitation outcomes [18]. Their work suggests that women may exhibit distinct immune and coagulation responses, potentially predisposing them to increased vulnerability under hypothermic stress. Supporting this, Solianik et al. demonstrated that although both sexes experience comparable rates of core temperature decline during cold exposure, women exhibit higher cortisol levels and divergent immunological responses—factors that may exacerbate the adverse effects of hypothermia [19]. These physiological differences warrant further investigation and should guide the development of sex-specific resuscitation strategies.

In our cohort, patients who were hypothermic upon ICU admission exhibited a threefold higher mortality risk compared to normothermic individuals. Those who developed hypothermia during early in-hospital care had a twofold increase in mortality (Table 2). Of note, the recently updated S3 Guideline *Intensive Care Medicine after Polytrauma* (German Interdisciplinary Association for Intensive Care and Emergency Medicine, August 2024) acknowledges hypothermia as a relevant risk factor and recommends routine body temperature monitoring as part of vital parameter assessment [20]. However, specific guidance on temperature management remains lacking. The observation that temperature data were incomplete in a substantial proportion of trauma cases may reflect a broader underrecognition of hypothermia’s clinical importance in routine practice.

Following traumatic injury, patients are exposed to significant thermal loss due to environmental exposure, diagnostic procedures, and surgical interventions. Several studies have demonstrated that active prehospital thermal management can significantly improve body temperature at the time of hospital admission [21]. During the early continuum of care—from ED resuscitation to ICU admission—forced-air warming systems have emerged as the most commonly used intervention, owing to their proven efficacy and cost-effectiveness [22]. Structured treatment algorithms such as the ABCDE approach advocate for early thermal protection as part of the primary survey, promoting consistency and standardisation in temperature management.

However, accurately quantifying the clinical impact of specific thermal interventions remains challenging due to numerous confounding variables and interindividual variability. At present, the literature does not provide sufficient evidence to support definitive recommendations. Importantly, the recent revision of the TR-DGU documentation protocol in 2025 has introduced the collection of detailed data on thermal management practices, which may facilitate more robust analyses and evidence-based guidance in future research [23].

This study is subject to several important limitations. Owing to the retrospective design of a registry analysis, certain details could not be fully captured. In particular, the TR-DGU does not provide information on the exact method used for body temperature measurement, which may introduce variability in the recorded values. Likewise, the registry does not provide information on which heat management strategies were applied in relation to the severity of hypothermia. Additional factors, such as ambient temperature, were not documented and therefore could not be considered in the analysis. A prospective, structured study design would allow these aspects to be systematically assessed and enable a more detailed evaluation of the advantages and disadvantages of specific approaches.

## Conclusion

In conclusion, hypothermia—particularly when persistent or newly acquired during early trauma care—emerges as a robust and independent predictor of adverse clinical outcomes, including increased mortality, coagulopathy, and transfusion requirements. Importantly, not only the presence of hypothermia at isolated time points but also dynamic changes in body temperature between ED admission and ICU transfer provide valuable prognostic insight. These findings underscore the clinical relevance of early temperature trajectories as a marker of systemic instability and highlight the necessity of timely, continuous thermal monitoring.

The data further support the implementation of proactive temperature management strategies as an integral component of acute trauma care. Early identification of patients at risk for thermal dysregulation—combined with evidence-based warming interventions—may contribute to improved outcomes, particularly in the critical window between initial resuscitation and definitive care.

Moreover, the observed sex-related disparities—specifically, the disproportionately higher incidence of hypothermia among female patients at ICU admission—raise important questions regarding sex-specific susceptibility to hypothermia and its physiological consequences. These findings warrant further investigation and may provide a rationale for the development of targeted temperature management protocols that account for sex as a biological variable in trauma resuscitation.

Collectively, our results emphasize that body temperature should not be viewed merely as a passive vital sign, but rather as an active, modifiable target in the resuscitative strategy for trauma patients. Standardised documentation and incorporation of temperature management into structured treatment pathways may help close the current gap between evidence and practice—ultimately improving outcomes in this vulnerable patient population.

## Data Availability

The data used in this study were obtained from the TR-DGU of the German Trauma Society. Access to the data is restricted to maintain patient confidentiality. Data can be made available from the corresponding author upon reasonable request and with permission from the TR-DGU.

## Abbreviations

AIS: Abbreviated Injury Scale
AUC AUC: Akademie der Unfallchirurgie GmbH
ED: Emergency department
GER/A/CH: Germany, Austria and Switzerland
ICU: Intensive care unit
ICM: Intensive care medicine
ISS: Injury Severity Score
OR: Operating room
Q: Quartiles
SD: Standard deviation
TR-DGU: TraumaRegister DGU^®^
PTT: Partial thromboplastin time
INR: International normalized ratio
